# Role of in silico structural modeling in predicting immunogenic neoepitopes for cancer vaccine development

**DOI:** 10.1172/jci.insight.136991

**Published:** 2020-09-03

**Authors:** Neeha Zaidi, Mariya Soban, Fangluo Chen, Heather Kinkead, Jocelyn Mathew, Mark Yarchoan, Todd D. Armstrong, Shozeb Haider, Elizabeth M. Jaffee

**Affiliations:** 1 The Sidney Kimmel Comprehensive Cancer Center, The Skip Viragh Center for Pancreatic Cancer, The Bloomberg–Kimmel Institute for Cancer Immunotherapy, The Johns Hopkins University School of Medicine, Baltimore, Maryland, USA.; 2 Department of Pharmaceutical and Biological Chemistry, University College London School of Pharmacy, London, United Kingdom.; 3 Department of Biochemistry, Faculty of Life Sciences, Aligarh Muslim University, Aligarh, India.

**Keywords:** Immunology, Oncology, Antigen

## Abstract

In prior studies, we delineated the landscape of neoantigens arising from nonsynonymous point mutations in a murine pancreatic cancer model, Panc02. We developed a peptide vaccine by targeting neoantigens predicted using a pipeline that incorporates the MHC binding algorithm NetMHC. The vaccine, when combined with immune checkpoint modulators, elicited a robust neoepitope-specific antitumor immune response and led to tumor clearance. However, only a small fraction of the predicted neoepitopes induced T cell immunity, similarly to that reported for neoantigen vaccines tested in clinical studies. While these studies have used binding affinities to MHC I as surrogates for T cell immunity, this approach does not include spatial information on the mutated residue that is crucial for TCR activation. Here, we investigate conformational alterations in and around the MHC binding groove induced by selected minimal neoepitopes, and we examine the influence of a given mutated residue as a function of its spatial position. We found that structural parameters, including the solvent-accessible surface area (SASA) of the neoepitope and the position and spatial configuration of the mutated residue within the sequence, can be used to improve the prediction of immunogenic neoepitopes for inclusion in cancer vaccines.

## Abstract 2

Structural parameters, including the solvent exposed surface area of the neoepitope and the position and spatial configuration of the mutated residue can be used to improve the prediction of immunogenic neoepitopes for inclusion in cancer vaccines.

## Introduction

The past decade has proven the therapeutic benefit of instructing the immune system to identify and kill cancer cells (1, 2). Immune checkpoint inhibitors (ICIs), such as anti–programmed cell death-1 (anti–PD-1), anti–programmed cell death ligand (anti–PD-L1), and anti–cytotoxic T lymphocyte–associated protein 4 (anti–CTLA-4) antibodies, are now a standard of care for some previously incurable cancers (3). These agents unleash the killing power of cancer-antigen–targeted effector T cells that infiltrate these immune responsive cancers. These T cells often recognize tumor-specific proteins called neoantigens that differentiate the tumor cell from its normal cell of origin. Neoantigens arise from nonsynonymous mutations, chromosomal rearrangements that result in fusion proteins, and/or insertions and deletions of bases into the genome (indels) during tumorigenesis (4, 5). They are presented to T cells by human leukocyte antigen (HLA) molecules on the tumor cell surface and are expressed only on tumors and not on normal cells. Certain tumors, such as melanomas and cancers with mismatch repair deficiencies (MMR-d) that lead to microsatellite instability (MSI), express a high burden of neoantigens (6, 7), whereas others, such as pancreatic cancers, glioblastomas, and MMR proficient (MMR-p) colon cancers, have modest to low neoantigen burdens (8–10). While the former group of tumors respond well to ICIs, the latter fail to induce and attract quality effector T cells within the tumor and, thus, respond poorly to single or dual agent ICIs (11).

One approach being developed to improve ICI responses against tumors with low mutational burden is to sequence the neoantigens in a given patient’s tumor and formulate a neoantigen-targeted vaccine to evoke high-quality, robust, neoepitope-specific T cell responses that can then be enhanced by ICIs. Such highly “personalized” vaccines have shown considerable promise in patients with hypermutated tumors such as melanoma. Patients with melanoma vaccinated with either synthetic long peptides (SLPs) (15–30 amino acids) or poly-neoepitope RNA (10 selected mutations within 27 amino acid peptides) displayed neoepitope-specific T cells in peripheral blood, which were shown to kill tumor cells expressing these neoantigens in vitro (12, 13). Two recent studies in patients with glioblastoma, a tumor type with a low neoantigen burden, reported the successful use of a personalized vaccine approach to stimulate neoantigen-specific T cell responses in peripheral blood. One patient was also noted to have T cells that trafficked into the tumor (9, 14). In a preclinical murine model of pancreatic cancer, we used a similar SLP-neoantigen–based vaccine approach to induce quality T cells that trafficked into a lower neoantigen burden tumor. We found that combination ICIs were also required to fully activate these tumor infiltrating T cells (TILs) to eradicate tumors (15).

The current pipeline for personalized vaccines in both preclinical and clinical settings uses whole exome sequencing (WES) and RNA sequencing (RNA-seq) to identify mutated proteins expressed in tumor tissue. An in silico algorithm, NetMHC, is then used to predict the binding affinity of each neoepitope to its MHC molecules. Those neoantigens with MHC binding affinities greater than 500 nM are predicted as being immunogenic and, hence, have been selected for inclusion in vaccines. However, in all 4 clinical studies (9, 12–14), only a small fraction of the neoantigens selected using MHC binding algorithms elicited robust neoantigen-specific T cell responses in the peripheral blood. Likewise, in our pancreatic cancer model, there was a discordance between MHC binding affinities as reported by NetMHC (versions 3.2, 3.4, and pan 2.8) and immunogenicity, as assessed by IFN-γ production by CD8^+^ T cells (2). Furthermore, there are instances where NetMHC-predicted neoepitopes have been noted to be functionally inhibitory (16).

A major challenge in further developing these personalized vaccines is the need to create algorithms that accurately select neoantigens expressed by a given patient’s tumor that are more likely to elicit the highest quality and most durable T cell responses. A recent study posits that the quality of neoantigens, noted for their similarity to pathogen-derived antigens known to elicit T cell responses, better predicts immunogenicity than T cell quantity alone (17, 18). Furthermore, structural modeling using existing crystal structures of HLA molecules suggest that the neoepitope, when presented at the MHC groove, must be solvent exposed to be recognized by the T cell receptor (TCR) (19). Of 170 neoepitopes predicted by NetMHC 3.4, 3.2, and pan 2.8 in a mouse colon cancer cell line (MC38), only 7 neoantigens were identified on mass spectrometry, of which 2 were immunogenic by qualitatively examining solvent exposure (19). We have defined immunogenicity narrowly as generating a positive response on our ELISPOT, with our readout showing IFN-γ–producing CD8^+^ T cells isolated from splenocytes vaccinated with respective neoepitope-corresponding peptides. More recently, there have been attempts to develop neural networks using the a general immune epitope database (IEDB), consisting mainly of viral antigens, by incorporating total binding energy scores from crystallographic and modeled structures (20). Here, we present a comprehensive structural evaluation in atom-level detail of how the solvent exposure and the configuration of the mutated residue within the MHC I grove facilitates its recognition by T cells.

## Results

### NetMHC predicts neoepitope immunogenicity inconsistently.

**Figure 1:**
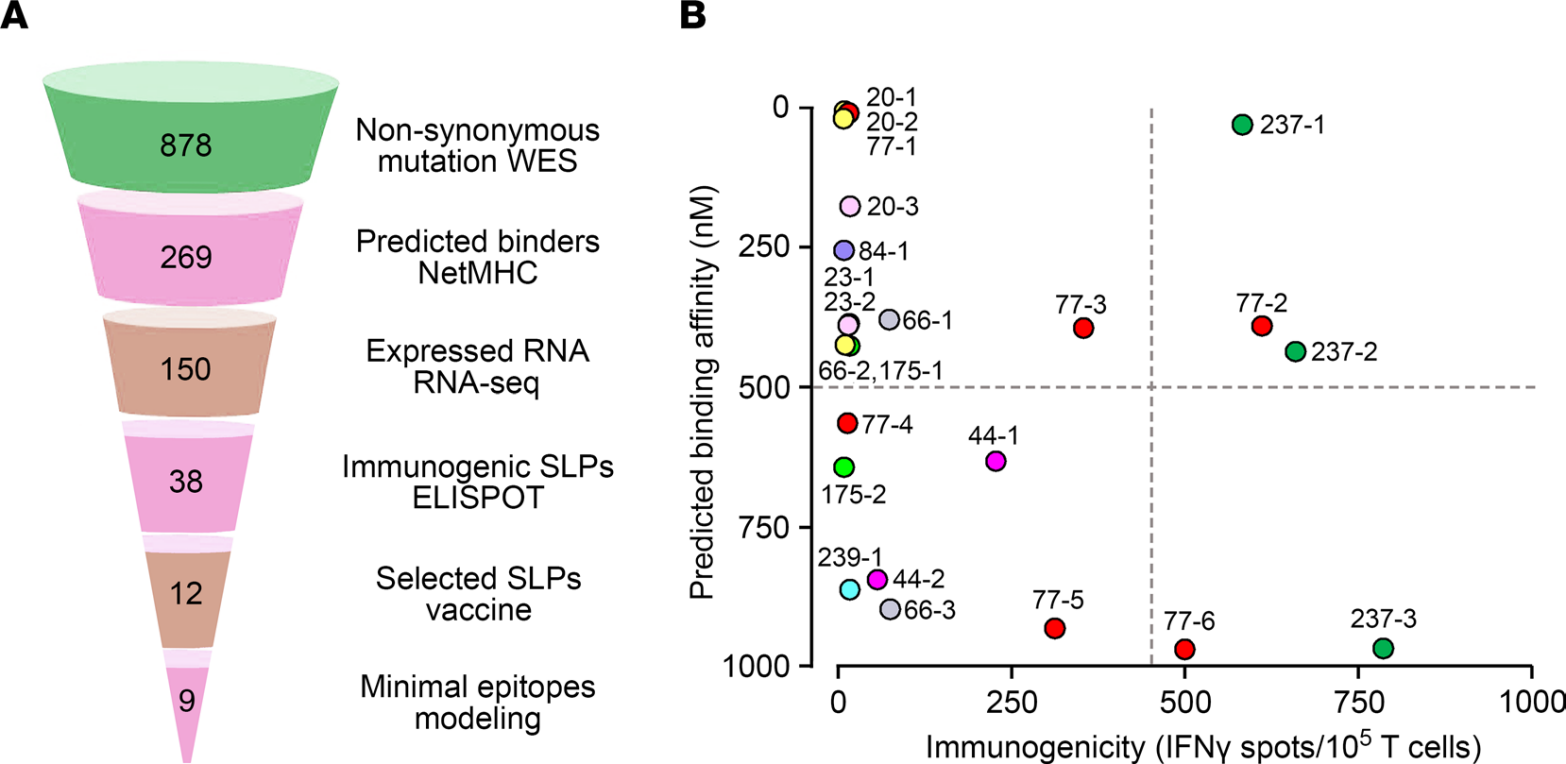
Identification of immunogenic tumor neoantigens in a murine pancreatic cancer model. (**A**) Pipeline for identifying nonsynonymous mutations in murine Panc02 cells by whole exome sequencing (WES); examining the transcriptome by RNA-seq; predicting binding affinity and minimal epitopes by NetMHC; generating synthetic long peptides (SLPs) for ELISPOT assays; and performing structural modeling on selected neoepitopes. For further details, please refer to ref. 15. (**B**) Inadequate correlation between predicted binding affinity (NetMHC 3.2, 3.4, and pan 2.8) of MEPs and their immunogenicity, as assessed by ELISPOT for IFN-γ–producing T cells in vitro (*n* = 3 mice per group) (for details on immunogenicity data, please refer to ref. 15). Note the clustering of high-affinity MEPs with poor immunogenicity (upper left quadrant).

We subjected the murine pancreatic Panc02 tumor to WES and identified 878 nonsynonymous mutations (Figure 1A). Two hundred and sixty-nine of these were predicted by NetMHC to bind either H-2Kb or H-2Db with affinities ≤ 1000 nM. NetMHC predicted 8– to 9–amino acid–long minimal epitope peptides (MEPs) for the neoantigens as potential vaccine candidates (Table 1). We directly tested immunogenicity in vitro using an ELISPOT assay for IFN-γ–producing CD8^+^ T cells. Figure 1B shows an imperfect correlation between the immunogenicity of individual MEPs and their predicted binding affinity derived from NetMHC. Certain MEPs, such as 20-3, 66-2, and 84-1, 175-1, and 175-2, were predicted to have high binding affinities stronger than 500 nM, a cut-off used in clinical studies (9, 12), but failed to elicit T cell responses. In contrast, MEP 237-3 was predicted to be a poor binder based on a lower binding affinity (<500 nM) but displayed the most potent immunogenic response. NetMHC correctly predicted the immunogenicity of certain variants, such as MEP 77-2 and 77-3. Thus, despite being widely used, the NetMHC-based algorithm does not consistently predict the immunogenicity of HLA/MHC binding MEPs.

### Structural templates influence neoepitope exposure.

The α1 and α2 subdomains of MHC fold to form a groove, with the curved base as a β-sheet and 2 α-helices separated at the top to accommodate 8– to 10–amino acid–long peptides. Therefore, we evaluated conformational alterations in and around the binding groove induced by selected minimal neoepitopes to understand the influence of a given mutated residue as a function of its spatial position.

**Figure 2:**
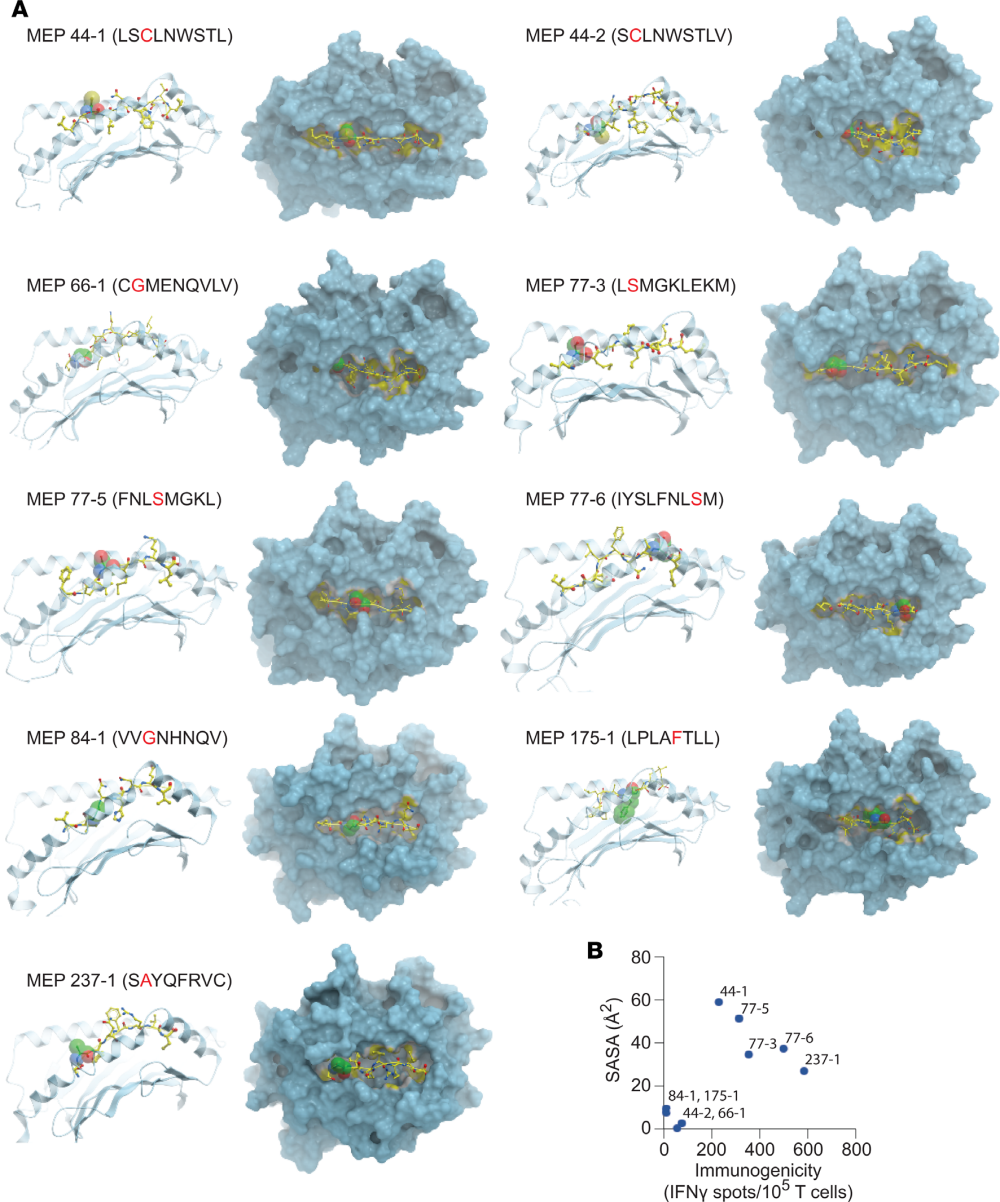
Modeling neoepitopes in selected MHC I templates. (**A**) Side (left panels) and top views (right panels) of modeled complexes between MEPs and appropriately selected MHC I template (also see Table 3, Table 4, and Table 5). MEP annotation and sequence are shown with the mutated residue in red. The ball represents the mutated residue and displays either an outward or inward orientation. This qualitative data are consistent with corresponding data from GETAREA that annotate the mutant residue and calculate the solvent-accessible surface area (SASA) (Table 3, Table 4, and Table 5). (**B**) Relationship between SASA (Å^2^) of the mutated residue of a given MEP and its immunogenicity, as assessed by ELISPOT for IFN-γ (for details on immunogenicity data, please refer to ref. 15).

We constructed multiple structural models of 9 MEP-HLA complexes (Figure 2A). Template-based homology modeling is based on the selection of templates with the highest sequence similarity to the modeled sequence. Alignment of our MEPs to the 40 H-2Db and 45 H-2Kb MHC I crystal structures available in PDB revealed sequence similarities between 33% and 87% (Table 2). We also found multiple templates in PDB that exhibited near-sequence similarities with a given MEP.

Structural modeling using existing crystal structures of HLA molecules suggest that neoepitopes must be exposed to solvent to be recognized by the TCR (19, 20). As we noted several high-sequence similarity–exhibiting structural templates for each MEP, each identified structural template needed to be inspected carefully for side chains that were either buried or exposed to solvent. For example, for MEP 44-1, which was immunogenic, we identified 5JWD as a high-sequence similarity template (at 55%) in which the mutated cysteine (Cys) residue side chain was solvent exposed (Table 2 and Figure 2A). Likewise, when modeling the nonimmunogenic 44-2 variant, 2ZOL was chosen at a 33% homology because the Cys side chain was in a buried state. For MEP 66-1, of the 5 identified templates, namely 1JPF, 3CCH, 3CH1, 3TBV, and 3TBW, we selected 1JPF with a sequence similarity of 55% (Table 2). For MEP 84-1, 4PG9 was selected with a sequence similarity of 75% from 4 templates, namely 1KPV, 2VAB, 4PG9, and 4PGC. For MEP 175-1, 7 templates (1KPV, 2VAB, 4PG9, 4PGC, 4PGD, 4PGB, and 4PGE) were identified, and 4PG9 was chosen with a sequence similarity of 62% (Table 2). For MEP 237-1, 2FO4 was selected with a sequence similarity of 87%. Three variants of MEP 77 were modeled (77-3, 77-5, and 77-6), in which the mutant Ser residue was solvent exposed, with 1BQH, ILK2, and 1S7R being chosen for the 3 variants (62%, 62%, and 50% homology, respectively). Overall, multiple templates based on epitope sequence homology were identified. The final template was chosen after taking into account whether the mutant residue was buried or exposed to solvent.

For initial testing, 8– to 9–amino acid–long epitopes containing the mutated residue, which had been previously tested for immunogenicity, were modeled into the MEP-HLA complexes using existing MHC I–peptide complex structures extracted from PDB as starting conformations (Figure 2A). To compare the modeled MEP-HLA complexes with the existing MEP-HLA complexes, we carried out an assessment of root square mean deviations (Cα RMSD), a measure of the average distance between atoms of docked proteins, for each optimized peptide with its corresponding template. The generally low Cα RMSD (0.75–1.33 Å) indicated that optimized complexes resembled the native complex conformation (Table 2).

### Mutated residue orientation and SASA predict immunogenicity.

Each modeled MEP-HLA complex was used to investigate the effect of the spatial position of the mutated residue, both qualitatively by examining buried (in) *versus* exposed (out) configurations (Figure 2A, top and side views), and quantitatively, by calculating solvent-accessible surface area (SASA) (Table 3, Table 4, and Table 5). Prior modeling of mutant MEPs in murine colon cancer suggested that mutated residues oriented toward the solvent interface were more likely to be immunogenic and that residue numbers 3–7 of any presented peptide were involved in TCR recognition (19). However, this may not always be the case, as an Ala residue at position 2 in MEP 237-1, which is solvent exposed, triggers immunogenicity (Table 5 and Figure 2A). Table 3, Table 4, and Table 5 list the contribution of different parameters calculated by the GETAREA module within FANTOM 4.0; this includes an automated output on whether the mutated residue is buried (in) or solvent-exposed (out). Of note, high SASA values (>20) noted with MEP 44-1, 77-3, 77-5, 77-6, and 237-1 showed an outward configuration of the mutated residue, whereas MEPs with low SASAs (<15), namely 44-2, 66-1, 175-1, and 84-1, displayed buried residues. Figure 2B shows that the high-SASA MEP-HLA complexes predicted good MEP immunogenicity, whereas those with low SASA values were less immunogenic.

### Shuffling mutated residues alters SASA, orientation, and immunogenicity.

Table 3, Table 4, and Table 5 show that, across the board, SASA values for each amino acid varied within MEP-HLA complexes. For example, the Leu residue next to the Ser in the MEP 77 models displayed SASAs of 3.32, 10.49, and 40.13 (Table 4). Hence, we posit that the spatial position of residues would determine SASA. A contribution of the spatial position to immunogenicity is best appreciated with MEP 44 (Table 3). The MEP 44-1–HLA complex with the mutated Cys residue at position 3, namely LS**C**LNWSTL, was constructed such that the side chain of Cys was solvent exposed (Figure 2A). The complex was confirmed by GETAREA to have a high SASA value (58.9) and an outwardly facing Cys side chain (Table 3). MEP 44-2 peptide also elicited a robust CD8^+^ T cell response on ELISPOT (Figure 1B). However, a shift of Cys by just 1 amino acid to position 2 (S**C**LNWSTLV) in MEP 44-2 lowered SASA by ~100-fold (to 0.51), buried the mutant Cys, and rendered the MEP less immunogenic on ELISPOT (Figure 2B).

To further test the hypothesis that the position of the mutated residue within a MEP can alter the orientation and SASA of that residue within the MHC I groove, we created 2 variants of MEP 77-5 by shifting the Ser residue by 1 amino acid. Notably, MEP 77-5 showed a low binding affinity on NetMHC (Figure 1B and Figure 2A). However, since it was immunogenic on ELISPOT and displayed an outwardly pointing Ser residue with a high SASA (51.27), we predicted that shuffling the position of the Ser residue within the MHC groove would alter SASA and residue orientation — and, therefore, immunogenicity. In the first sequence, the mutated Ser residue was shuffled from fourth to fifth position (FNL**S**MGKL → LFNL**S**MGK) (termed MEP 77-5v1) (Table 6). In the second sequence, the Ser residue was shuffled from the fourth to the sixth position (FNL**S**MGKL → SLFNL**S**MGK) (termed MEP 77-5v2).

**Figure 3:**
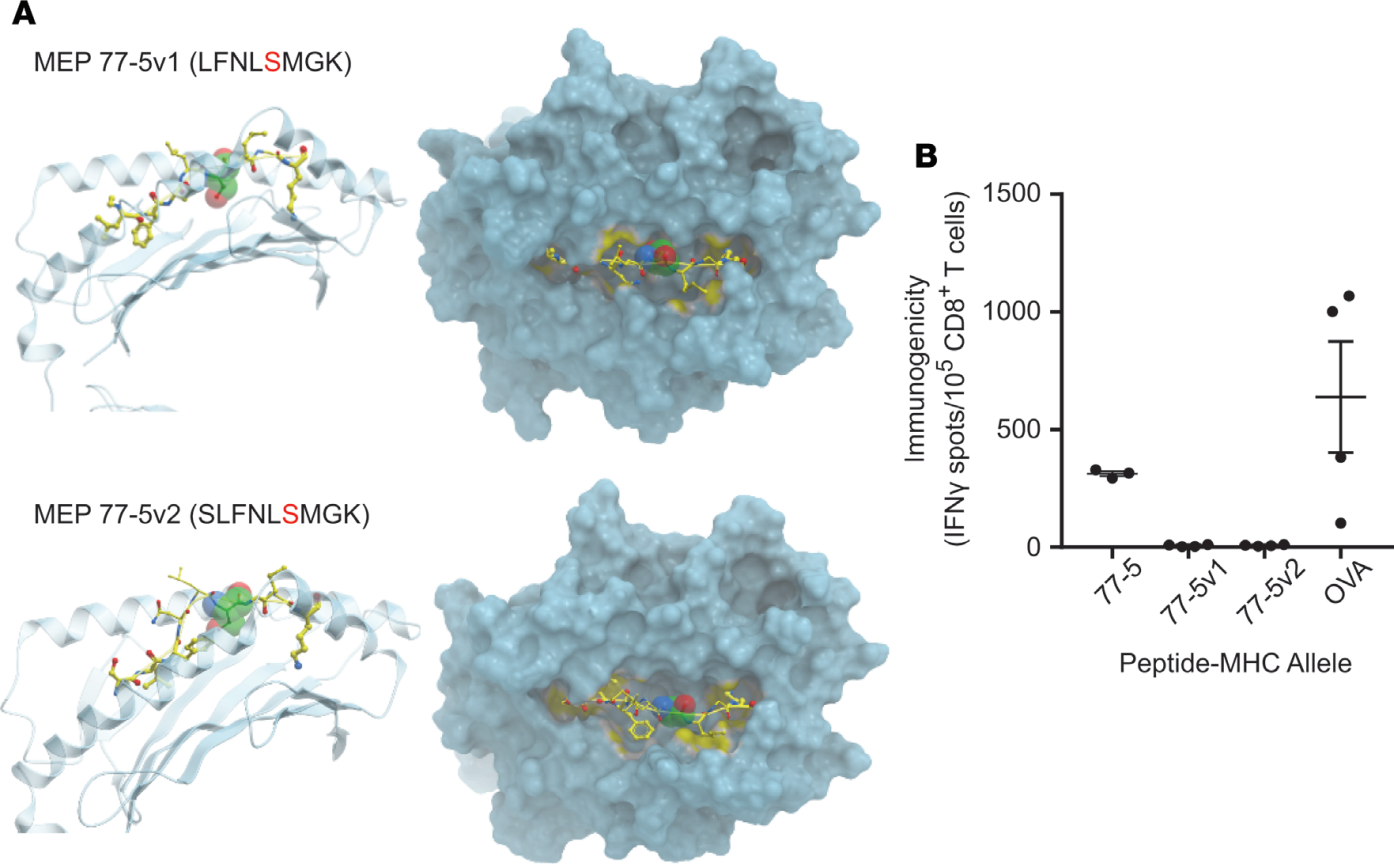
β-Testing of MEP variants for SASA and immunogenicity. The position of the Ser residue in MEP 77-5 was shifted by 1 amino acid in 2 variant models, MEP 77-5v1 (FNL**S**MGKL → LFNL**S**MGK) and MEP 77-5v2 (FNL**S**MGKL → SLFNL**S**MGK). (**A**) Side (left panels) and top views (right panels) of modeled complexes between MEPs and selected MHC I template. The ball represents the mutated residue. Calculated SASA and other GETAREA parameters are shown in Table 4. (**B**) Immunogenicity of variant MEPs by ELISPOT for IFN-γ–producing T cells. An H-2Kb–binding OVA (OVA-Kb) peptide is used as control (for details, see Methods) (*n* = 3 mice per group).

For MEP 77-5v1, 3 templates were identified (1G7P, 1VAD, 1G7Q), of which 1G7P was selected exhibiting 50% similarity. MEP 77-5v2 identified 4 templates (1S7Q, 1S7R, 1S7S, 1S7T), from which 1S7T was selected with 50% similarity (Figure 3A and Table 6). GETAREA analysis of the variant MEP-HLA models revealed low SASAs and buried Ser side chains. Both variant peptides also displayed poor immunogenicity on ELISPOT assays (Figure 3B). Thus, shuffling the mutated residue by 1 and 2 positions in the original MEP 77-5 significantly attenuated immunogenicity. In all, both post hoc and a priori modeling testify to the critical roles of SASA and mutant residue orientation and position in predicting the immunogenicity of minimal neoepitopes.

## Discussion

We studied the presentation of 9 predicted neoepitopes used in our pancreatic cancer vaccine PancVAX (15) by modeling these onto MHC I templates derived from crystal structures from the Protein Data Base (PDB). Much more complex than previously envisioned (19), we show that the prediction of cancer neoantigen immunogenicity by in silico modeling (a) depends upon the selected template, (b) correlates with the SASA of the neoepitope, and (c) can be modified by shuffling the mutated residue within the sequence, which, in turn, exposes or buries the single mutated residue. This atomistic detail provides the framework for incorporating 3D structural parameters into automated machine learning algorithms as a high-throughput neoepitope prediction tool of the future.

For effective personalized cancer vaccines, it is imperative that nonsynonymous mutations arising during tumorigenesis not only yield expressed antigens that are processed and presented, but that the neoantigens that are included in a vaccine are able to trigger a robust and durable T cell response. In our Panc02 model, we have effectively demonstrated a biologically meaningful response after vaccination with PancVAX by demonstrating both reduced tumor growth and induction of neoantigen-specific T cells within the tumor (15). Following WES, which identifies DNA variants, RNA-seq and, in some instances, mass spectrometry, have been used to identify the DNA variants that are tumor-expressed proteins and neoepitopes presented on MHC (2, 9, 12–14). However, it has been much more challenging to determine which expressed neoantigens evoke a neoepitope-specific T cell response within tumor tissue and in peripheral blood.

Recent studies have used NetMHC to determine binding affinities of expressed neoantigens to HLA and MHC I as surrogate readouts for T cell immunity. An algorithm based on artificial neural networks, NetMHC, is trained for 81 different human HLA alleles and 41 animal MHC I alleles, which include murine MHCs (21, 22). The algorithm has been used widely to predict MHC-peptide binding for 8-, 10-, or 11-amino acid peptides (using 9–amino acid trained predictors) in several pathogenic viral proteomes, including SARS, influenza, HIV, and hepatitis C (23, 24). This training has resulted in 75%–80% confirmed HLA/MHC I binders, with further validation and benchmarking using large sets of nonredundant affinity data (22–24). However, in the case of tumor neoantigens, NetMHC binding has only inconsistently predicted an immune response (2, 9, 12–14) (Figure 1B). In both glioblastoma and melanoma trials, patients were vaccinated with up to 20 SLPs based on NetMHC predictions. In the melanoma studies, only 2–4 SLPs evoked a neoepitope-specific CD8^+^ T cell response in the peripheral blood, whereas in the glioblastoma study, only 1 patient generated a CD8^+^ T cell response to 2 neoepitopes. Likewise, in our study with murine pancreatic cancer, Figure 1B illustrates a cluster of high-binding MEPs, such as 20-1, 20-2, and 77-1, among others, that display minimal or no immunogenicity.

The inconsistent prediction of neoepitope immunogenicity may indeed arise from the fact that NetMHC is trained to enact binding affinities of foreign pathogenic antigens, not tumor neoantigens. Specifically, it may not be trained well enough to distinguish as precisely between tumor neoantigens arising from point mutations with just a single amino acid difference. This apparent discrepancy will then be consistent with the “quality model,” in which high-quality neoantigens are considered to rank closer to pathogen-derived peptides (17, 18). In this instance, it would also be biologically meaningful to hypothesize that the mutated residue must be prominently exposed to solvent at the MHC I groove to be detected as foreign by the TCR (19).

We tested this hypothesis by structural modeling to calculate specifically a well-characterized parameter for solvent exposure, SASA (25). SASA has been used widely to evaluate the structure, function, and stability of proteins. Its uses have ranged from drug design and discovery, to studies on the protein-DNA binding interfaces, to understanding phylogenetic coevolution of proteins (26, 27). Here, we present a comprehensive SASA-based structural analysis of MEP-HLA complexes to further inform the immunogenicity of a given neoantigen before its inclusion in a vaccine. Importantly, the automated calculation of SASA by GETAREA is also accompanied by “in” or “out” annotation on orientation of the mutant residue — overall providing a comprehensive readout for solvent exposure

Furthermore, and to a higher level of atomistic detail, our prespecified modeling and β-testing both show that minor changes in the positioning of mutated residue within the MEP can have a profound effect on T cell recognition and immunogenicity. While for MEP 44-2 (LS**C**LNWSTL), the complex displayed a high SASA, a solvent-exposed Cys side chain, and a robust CD8^+^ T cell response, a shift of the Cys residue by just 1 amino acid (S**C**LNWSTLV) in MEP 44-2 buried the Cys, reduced SASA by ~100-fold, and rendered the MEP less immunogenic. In our β-testing protocol, we created 2 variants of immunogenically responsive MEP 77-5, FNL**S**MGKL → LFNL**S**MGK and FNL**S**MGKL → SLFNL**S**MGK, by shifting the mutated Ser from position 4 to position 5 or 6 in the 8-mer. Both a single and 2–amino-acid shuffle reduced SASA, buried the Ser residue, and diminished immunogenicity. That these atom-level modifications alter immunogenicity would again make it unlikely for current versions of NetMHC, to detect subtle differences in the position of the mutant residue at the MHC I groove, without further training on 3D events.

A recent open-source package, MHCflurry, has been presented as a more accurate predictor of MHC I binding compared with 2 publicly available algorithms, NetMHC_4.0_ and NetMHC_pan_, particularly for non-9 amino acid peptides (i.e., peptides of lengths 8 and 10–15) and portends a ~400-fold higher speed (28). It is different from the NetMHC versions in that it uses a publicly trainable architecture and mass spectrometry–identified peptides for model selection (28). While we await more data on outcomes from the new algorithm, it is unclear whether these algorithms can be trained to seek information on protein structure. Given our data, we find that any new module or automated learning machine to more accurately predict neoantigen immunogenicity must have access to PDB and be able to extract the best template. A major challenge is likely to be centered on the selection of the correct template from several high-sequence homology templates for each minimal epitope.

## Methods

### Neoantigen identification pipeline.

Extracted DNA and RNA from a murine pancreatic cancer cell line Panc02 (29) was subject to WES and RNA-seq, respectively. The raw sequencing files from WES were aligned to the mm9 reference genome using bowtie2. Variants were called using freebayes and subsequently annotated using Annovar (2). Tumor-specific peptide sequences were extracted using R and analyzed by locally maintained NetMHC 3.2, 3.4, and pan 2.8 algorithms (https://github.com/rosgood/Panc02_Variant_ID/commit/899bdd743f671822ef17749769d100ae7f65e698) (2). All mutated epitopes predicted to bind to H-2Kb or H-2Db with an affinity of ≤ 1000 nM were manually curated by Integrated Genomics Viewer. Pipeline is available on git repository at https://github.com/rosgood/Panc02_Variant_ID/commit/899bdd743f671822ef17749769d100ae7f65e698 For neoepitopes with predictions of ≤ 1000 nM, corresponding 20-mer peptides with mutations centered at position 11 were synthesized initially at 70% purity by Peptide 2.0. Further details are available in ref. 15.

### Immunogenicity testing.

Groups of 6-week-old male C57BL/6 mice (3–4 mice per group) each received a pool of 5–6 SLPs at 50 μg per peptide, the known H-2Kb–binding OVA (amino acid positions 152–171) peptide GLEQLESIINFEKLTEWTSS (10 μg), and poly IC:LC (10 μg) (InVivoGen). An initial injection of the vaccine into the hind limb was followed by a boost at day 7 and IFN-γ ELISPOT at day 13 to evaluate CD8^+^ T cell responses (30). We also mapped the minimal epitopes of the respective 20-mers by similarly testing 8– to 11–amino acid–long peptides corresponding to the expected minimal immunogenic epitopes as predicted by NetMHC to have binding affinities of ≤ 1000 nM (15).

### T cell isolation and ELISPOT.

CD8^+^ T cells were isolated from freshly harvested splenocytes by first creating a single-cell suspension by passing the spleen through a 40-μm filter in CTL media (RPMI with 10% FBS [Thermo Fisher Scientific], 0.5% L-glutamine, 1% penicillin/streptomycin [Invitrogen], and 0.05 mM 2-mercaptoethanol [Thermo Fisher Scientific]). Erythrocytes were removed by ACK lysis, and the resulting T cells were washed, counted, and isolated using the Dynal CD8^+^ negative isolation kit (Dynal, Invitrogen) per manufacturer’s instructions. Multiscreen 96-well filtration plates (MilliporeSigma) were coated overnight at 4°C with 100 μL/well of anti–mouse IFN-γ mAb AN18 (100 μg/mL, Mabtech, catalog 3321-3-250). Wells were washed 3 times each with PBS and blocked for 2 hours with CTL media at 37°C. A total of 1 × 10^5^ T2 APCs was pulsed with 2 μg peptide in 100 μL CTL media for 2 hours at 37°C, 5% CO_2_. After T cell isolation, 1 × 10^5^ CD8^+^ T cells were added to the capture plate in 100 μL CTL, followed by the addition of T2 APCs with peptide and incubation for 18 hours at 37°C at 5% CO_2_. Cells were removed from the plate by washing 6 times, 2 minutes per wash, with PBS plus 0.05% Tween-20 (MilliporeSigma). Wells were incubated for 2 hours at room temperature with 10 μg/mL biotinylated anti–mouse IFN-γ mAb R4-6A2 (Mabtech, catalog 3321-6-250) in 0.05% FBS diluted in PBS. Wells were washed as before, incubated with avidin peroxidase complex (Vectastain ELITE ABS kit; Vector Laboratories) for 1 hour at room temperature, and washed again. AEC substrate was added, and wells were developed for 10–15 minutes at room temperature. The reaction was stopped with tap water, and plates were allowed to dry for 24 hours before they were counted using an automated image ELISPOT reader (ImmunoSpot).

### Structural modeling.

To understand the structural influence of the mutated residues in the MEP as a function of its spatial position and configuration, we constructed multiple MEP-HLA complexes. The template structures to model the peptides were identified by searching the PDB using PSI-BLAST (31) for their corresponding homolog in H-2Kb and H-2Db structural sets. MEP 44 and 66 were modeled as MHC I H-2Db, while MEP 77, 84, 175, and 237 were modeled as MHC I MHC I H-2Kb complexes. Hits that exhibited highest sequence identity were selected. The MEP and template sequences were aligned using ClustalX (32). The final template was chosen from the selected set, wherein the mutated residue was solvent exposed.

We generated models of each MEP using MODELLER v9.20 (33) and evaluated stereochemical properties using PROCHECK (34) and ProSA (35). Multiple models were generated, with a final model being chosen based on low-energy function and a low Cα RMSD overlap between the template and the modeled MEP. The peptide in the protein-peptide complex was then optimized using a Rossetta-based algorithm implemented in the FlexPepDock server (36). In short, the starting structure was refined in 200 independent FlexPepDock simulations. Of these, one half was run in the high-resolution model, while the other 100 included a low-resolution preoptimization step, followed by high-resolution refinement. Models generated were then ranked based on their Rosetta generic full-atom energy score. The final model was selected based on solvent accessibility of the mutated residue in the MEP and the lowest energy score from the FlexPepDock optimization.

### Derivative measures.

We derived SASA of each residue of a given MEP using GETAREA, a program implemented within FANTOM (http://curie.utmb.edu/getarea.html). In addition to SASAs, this calculation yielded atomic solvation energies and their gradients for the macromolecules. Briefly, the server accepts PDB coordinates as an input file and reports several outputs, including total surface area, apolarlty, and contribution of the backbone and side chains in a postprocessed PDB file (Table 3, Table 4, and Table 5). Furthermore, an indication of whether the amino acid is buried (in) or solvent exposed (out) is annotated for each residue in the PDB file (Table 3, Table 4, and Table 5). All structural calculations and figures were generated using ICM-Pro software (25).

### Study approval.

All animal studies were reviewed and approved by the Johns Hopkins IACUC and Biohazards Committee. Animals were kept in pathogen-free conditions and were treated in accordance with institutional and American Association of Laboratory Committee policies. All efforts were made to limit animal pain and discomfort.

## Author contributions

Study conception and design were contributed by NZ, SH, and EMJ; acquisition of data was contributed by NZ, MS, FC, HK, JM, MY, TDA, and SH; analysis and interpretation of data were contributed by NZ, MS, FC, HK, and SH; drafting of manuscript was contributed by NZ, SH, and EMJ; study supervision and critical revision were contributed by EMJ, SH, and NZ; and all authors read and approved the manuscript.

## Figures and Tables

**Table 1:**
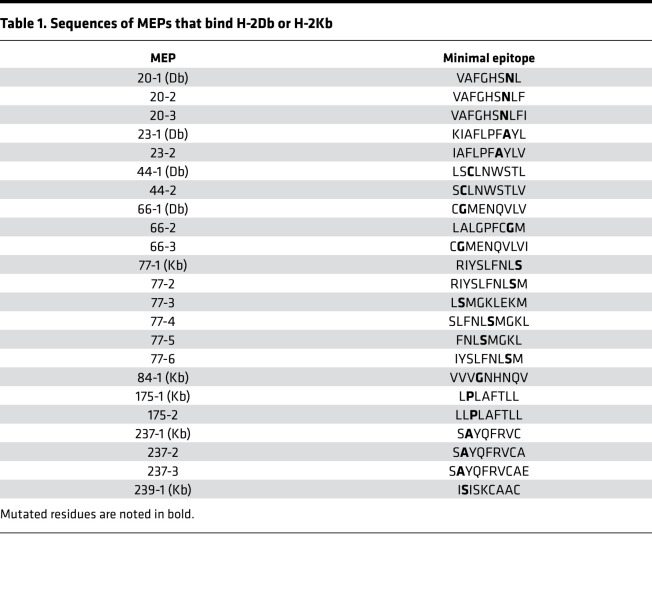
Sequences of MEPs that bind H-2Db or H-2Kb

**Table 2:**
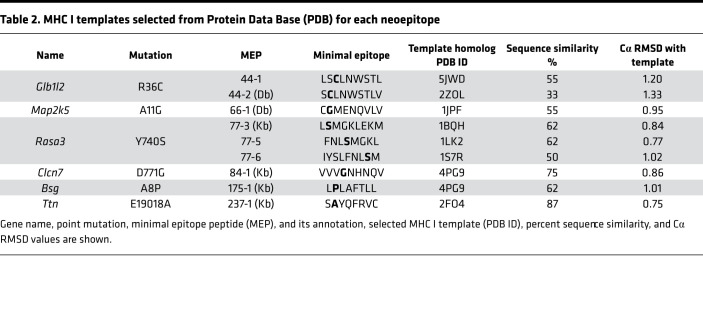
MHC I templates selected from Protein Data Base (PDB) for each neoepitope

**Table 3:**
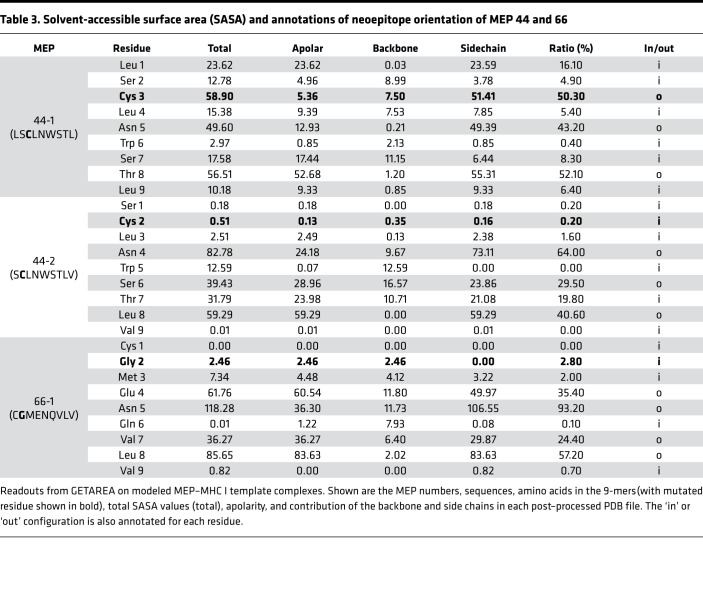
Solvent-accessible surface area (SASA) and annotations of neoepitope orientation of MEP 44 and 66

**Table 4:**
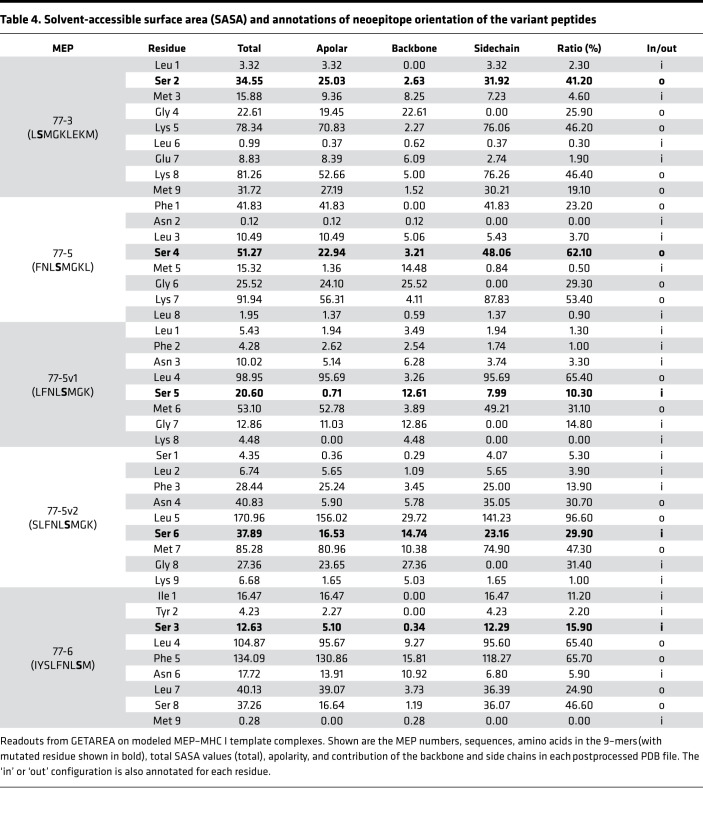
Solvent-accessible surface area (SASA) and annotations of neoepitope orientation of the variant peptides

**Table 5:**
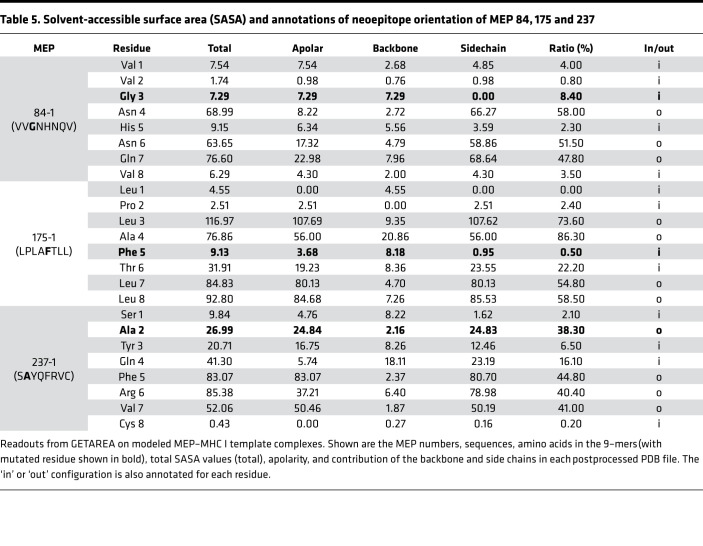
Solvent-accessible surface area (SASA) and annotations of neoepitope orientation of MEP 84, 175 and 237

**Table 6:**
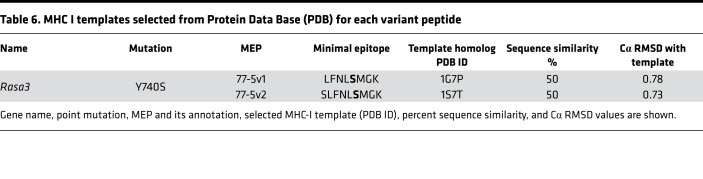
MHC I templates selected from Protein Data Base (PDB) for each variant peptide

## Version 1 09/03/2020

Electronic publication
